# Ruptured Pseudoaneurysm With Catastrophic Hemorrhage

**DOI:** 10.7759/cureus.112122

**Published:** 2026-07-06

**Authors:** Kenza Benzmane, Hanane Boumlik, Lahcen Kaddouri, Youssef Belchiti, Abdelkarim Kharroubi

**Affiliations:** 1 Vascular Surgery, University Hospital Center (CHU) Souss Massa, Agadir, MAR; 2 Medicine and Pharmacy, Université Ibn Zohr, Agadir, MAR

**Keywords:** catheterization of a fistula, hemodialysis vascular access, hemorrhage, pseudoaneurysm, vascular access complications

## Abstract

Hemodialysis sessions require a vascular access that can be used frequently and allows blood flow rates of at least 350 mL/min. The creation of an arteriovenous fistula makes the vein easily puncturable; it is the main choice because of its longevity. Some complications can lead to death. Knowing them can prevent the worst-case scenario during the hemodialysis session or afterward. We report an unusual complication of initial arteriovenous access cannulation that causes a pseudoaneurysm of the brachial artery and is treated surgically. Treatment options for pseudoaneurysm management include endovascular treatment and surgical repair.

## Introduction

We present a case of a young boy with an extremely rare complication of hemodialysis. An unsuccessful venous cannulation of the arteriovenous fistula caused a brachial artery pseudoaneurysm. The arteriovenous fistula (AVF) is a communication between a native artery and vein, used as a hemodialysis access due to its lower complication rate and longer longevity compared with other access options. Nevertheless, some complications can be noted, in particular stenosis, thrombosis, arm edema, steal syndrome, ischemic neuropathy, infection, aneurysms, and pseudoaneurysms [1]. Pseudoaneurysms, also known as false aneurysms, are anomalies of the arterial wall, especially after inadvertent trauma. Clinically, the pseudoaneurysm presents as a pulsatile mass that gradually increases in volume with a risk of rupture [2]. This complication must be prevented and avoided by medical personnel.

## Case presentation

A 13-year-old male was referred to our institution from a secondary hospital immediately after bleeding from his radiocephalic arteriovenous fistula with a compressive bandage and a tourniquet. The hemodialysis fistula had been in place for two months. One week earlier, he had been admitted for venous cannulation before his first hemodialysis session. A progressively larger mass had developed at the antecubital fossa. On first examination, the patient was hemodynamically stable, and the bleeding had stopped. He presented with a nonpulsatile mass, elbow stiffness, dermal tissue necrosis, and edema of the left hand (Figure 1).

**Figure 1:**
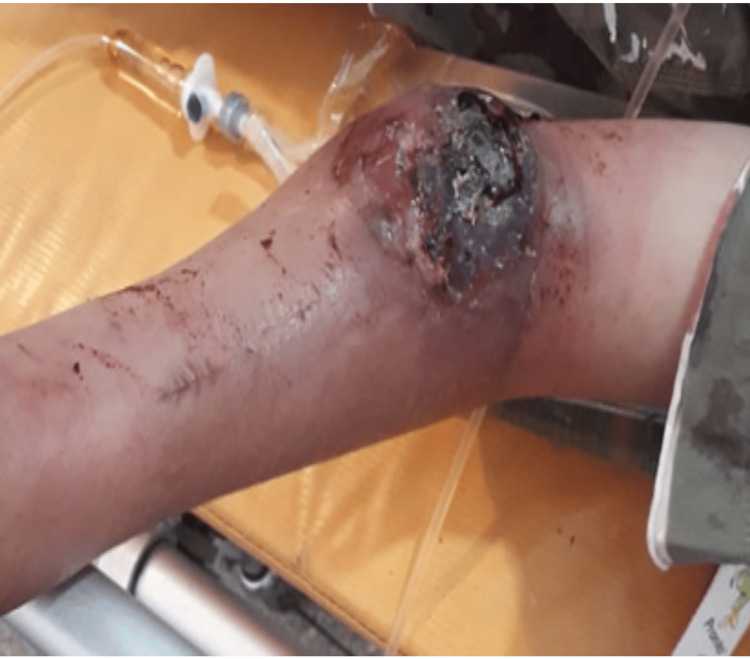
Pre-operative appearance showing a mass in the left elbow and dermal tissue necrosis.

The pulses in the arm were palpable, and the arteriovenous fistula thrill was present. His hemoglobin level was 5.8 g/dL. Doppler ultrasonography revealed a pseudoaneurysm arising from a defect in the anterior wall of the left brachial artery in the antecubital fossa and an approximately 7-cm hematoma compressing the veins. The decision to perform surgery was made after correcting his anemia. The patient was admitted to a session of hemodialysis with a tunneled central venous catheter, and a transfusion was performed. During the session, the bleeding started, and the patient was taken urgently to the operating room.

The surgery was performed under a tourniquet in order to limit the bleeding during the procedure. General anesthesia was administered. The hematoma was removed, and the brachial artery was exposed. The arterial puncture site was identified, and hemostasis was achieved with a 7-0 Prolene suture (Figures 2a-2d). The arteriovenous fistula was preserved. The post-operative course was uneventful, and the edema decreased.

**Figure 2:**
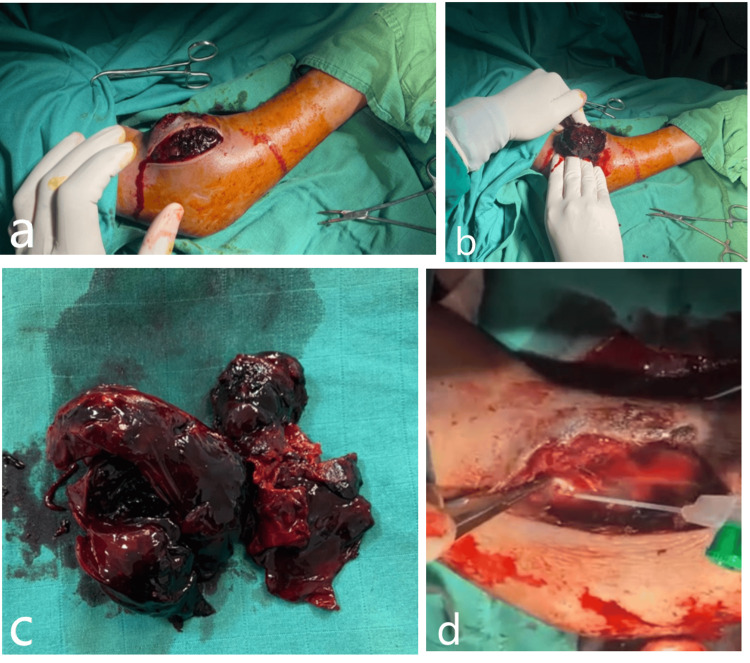
Intra-operative steps during evacuation of the hematoma and repair of the brachial artery. (a) Cutaneous incision showing a large hematoma. (b) Removal of the hematoma. (c) Excised hematoma (surgical specimen). (d) Puncture site of the brachial artery after exposure.

## Discussion

Before every hemodialysis session, the arteriovenous (AV) access should be monitored for the main venous outflow and thrill to assess for infection, steal syndrome, or aneurysm/pseudoaneurysm. In case of a primary fistula and first cannulation, the fistula must be mature. For that, the rule of 6s should be respected as follows: blood flow greater than 600 mL/min, vessel diameter of at least 6 mm, depth of no more than 6 mm, an interval of at least 6 weeks between creation of the fistula and cannulation, and a distance of at least 6 cm between the two needles.

The most common causes are trauma and iatrogenic injuries. A pseudoaneurysm of a hemodialysis arteriovenous fistula (AVF) occurs in 0.01-2% of cases [3]. The initial AV access cannulations must be performed by skilled cannulators who adhere to the guidelines to minimize the risk. Signs on physical examination that require urgent intervention include thin, shiny, depigmented skin; ulcers or scabs; and extensive infiltration. There are the following three types of cannulation injuries: minor, major, and severe (Table 1) [4].

**Table 1:** Cannulation injury for hemodialysis AVF. AVF: arteriovenous fistula

The type of injury	Clinical examination	Treatment
Minor cannulation injury	Bleeding, infiltration, swelling	Conservative management (ice and rest for 1 to 2 days). Cannulation can be done at the next dialysis session
Major cannulation injury	Significant bleeding, infiltration, and swelling	Rest for >7 days
Severe cannulation injury	Bleeding complications	Requires: hospitalization, blood transfusion, endovascular or surgical intervention

This injury must be known to the hemodialysis staff to prevent issues. The main complications are death by hemorrhage and compression signs [4]. Risk factors related to this anomaly are obesity, use of anticoagulants and antiplatelet agents, hemodialysis, peripheral vascular disease, hypertension, large sheath size, puncture technique, short manual compression, and catheterization of both arteries and veins [5,6].

The gold standard for diagnosis of iatrogenic pseudoaneurysms is color duplex ultrasound; the evaluation should visualize the vessels, the sac size with flow, the neck diameter, and the color flow signal in a tract leading to a sac [7]. Also, angiography confirms the lesion and its localization.

Three main treatment options are proposed, depending on the cause and presence of complications. The surgical repair is indicated when symptomatic pseudoaneurysms are found, such as expanding hematoma, infection, or compressive signs. Ultrasound-guided compression consists of finding the neck leading into the pseudoaneurysm, and pressure is applied for 10 min until the flow decreases. Finally, endovascular methods treat the pseudoaneurysm with embolization or stent grafts [5].

In the literature, brachial artery injuries in hemodialysis patients after cannulation of their surgically created AVF are rare. The majority of reported cases are related to venous pseudoaneurysms of the arteriovenous fistula rather than the much rarer complication of brachial artery injury [8-10]. Table 2 provides a summary of these documented cases.

**Table 2:** Published case reports and series of arterial complications associated with hemodialysis arteriovenous fistula. AVF: arteriovenous fistula

Studies	Type of lesion	AVF context	Number of cases	Main treatment
Sarılar et al. (2022) [11]	Ruptured pseudoaneurysm	Accidental arterial puncture during dialysis	1	Surgical repair
Rahman et al. (2024) [12]	Giant pseudoaneurysm	After repeated fistulograms	1	Excision + arterial patch angioplasty
Cox et al. (2015) [13]	Pseudoaneurysm post-ligation	AVF ligated 14 years prior	1	Excision + end-to-end anastomosis
Soares et al. (2017) [14]	True aneurysm (long term)	20 years after AVF creation	5 + review of 21 cases	Vein graft or arterial transposition
Allinjawi et al. (2019) [15]	Multiple aneurysms	Anatomical variation	2	Vein graft or ligation
Ferrara et al. (2016) [16]	Giant true aneurysm	Post-spontaneous AVF closure	1	Excision + vein graft

## Conclusions

Hemodialysis replaces the function of the kidney when it fails. It allows patients with kidney failure to survive by removing waste products and excess fluid from the blood. For this reason, vascular access is important. Initial arteriovenous access cannulations must be performed by skilled technicians, nurses, or doctors. Also, the medical staff must be aware of the complications so they can be treated early.
